# CGP 35348, GABA_**B**_ Receptor Antagonist, Has a Potential to Improve Neuromuscular Coordination and Spatial Learning in Albino Mouse following Neonatal Brain Damage

**DOI:** 10.1155/2014/295215

**Published:** 2014-04-06

**Authors:** Q. Gillani, M. Ali, F. Iqbal

**Affiliations:** ^1^Zoology Division, Institute of Pure and Applied Biology, Bahauddin Zakariya University, Multan 60800, Pakistan; ^2^Institute of Molecular Biology and Biotechnology, Bahauddin Zakariya University, Multan 60800, Pakistan

## Abstract

To study the effect of CGP 35348 on learning and memory in albino mice following hypoxia ischemia insult, 10 days old albino mice were subjected to right common carotid artery ligation followed by 8% hypoxia for 25 minutes. Following brain damage, mice were fed on normal rodent diet till they were 13 week old. At this time point, mice were divided into two groups. Group 1 received saline and group 2 intrapertoneally CGP 35348 (1 mg/mL solvent/Kg body weight) for 12 days. A battery of tests used to assess long term neurofunction (Morris water maze, Rota rod and open field) along with brain infarct measurement. Overall CGP 35348 has improved the motor function in male and female albino mice but effects were more pronounced in female albino mice. In open field, CGP 35348 treated female albino mice had demonstrated poor exploratory behavior. During Morris water maze test, gender specific effects were observed as CGP 35348 had improved spatial learning and memory and swimming speed in male albino mice but had no effect in female albino mice following hypoxia ischemia encephalopathy (HIE). We concluded that GABAB receptor antagonists CGP 35348 can be used to improve gender based spatial memory.

## 1. Introduction

Oxygen deprivation in any part of the body is termed as hypoxia. In a context of neurology, it is reduced oxygen supply to brain despite adequate amounts of blood supply [1, 2]. During gestation or delivery the accidental asphyxia of infants or perinatal hypoxia ischemia (HI) is a common cause of neurological deficits and delayed cognitive and behavioral deficits [3]. In the first few weeks these infants develop diffuse white matter injury rather than acute gray matter loss [4] but it leads to long-term dysfunction of both gray and white matter structures [5].

GABA_B_ receptors are heterodimeric G protein-coupled sites.  GABA_B_  are located both pre- and postsynaptically [6]. GABA_B_ receptor antagonist's experiments on laboratory animals, both rodents and primates, suggest that it enhances learning and memory but these effects might vary under different conditions [7]. CGP 35348 is antagonist with significantly higher affinity for post-versus presynaptic receptors [8] that crosses the blood-brain barrier [9]. CGP 35348 administration increased glutamate release but not that of GABA [10].

To our knowledge, till now there is not a single report regarding the potential role of GABA_B_ receptor antagonist in learning and memory improvement following brain damage. The present study was designed to compare the effects of GCP 35348 on neurological test battery performance and brain infract volume between GABA_B_ treated and untreated male and female albino mice following hypoxic ischemic insult.

## 2. Materials and Methods

### 2.1. Subject

Albino mice [both male (*N* = 20) and female (*N* = 20)] were used in the present study. Mice were maintained in locally manufactured cages provided with wood chips at the core animal facility in Bio park of Bahauddin Zakariya University, Multan. Albino mice were kept in separate cages with free access to water and standard rodent diet throughout the experimental duration. Room temperature was maintained at 22 ± 1°C and relative humidity was 50 ± 10%. The light/dark rhythm was 14 : 10. All the experimental procedures and mouse handling protocols were approved by the ethical committee of Institute of Pure and Applied Biology, Bahauddin Zakariya University, Multan.

### 2.2. Murine Model of Hypoxia Ischemia Encephalopathy

On postnatal day 10 of pups, corresponding to the brain development of 40-week gestational age in human fetus [11], pups were anesthetized with isoflurane (3%) inhalation. A right lateral neck incision was made and the right common carotid artery was ligated using polypropylenedalcon USP 6 suture. Pups were kept on a hot plate with constant 36°C temperature during the surgery. The entire surgical procedure was completed within 10 min after which pups were allowed to recover and nurse for 30 min with their dams. Mice were then placed for 25 min in a hypoxic chamber with constant flow of 8% oxygen balanced with nitrogen. The hypoxic chamber was kept on hot plate to maintain the ambient temperature inside the chamber at 36°C. Following hypoxic exposure, pups were kept with their mothers for recovery. Following weaning on postnatal day 20, mice were separated from their parents and housed in individual cages.

### 2.3. Experimental Design

Following weaning, mice were separated from their parents and fed on normal mouse diet until 13th week of life when they either received intraperitoneal injections of GABA_B_ receptor antagonist CGP 35348 [(3-aminopropyl), (diethoxymethyl) phosphinic acid] at the rate of 1 mg/mL solvent/Kg body weight or saline solution for 12 days. CGP 35348 was dissolved in saline solution.

### 2.4. Rotarod

Balance and coordination in mouse was observed by using rotarod (locally manufactured) tests. It consists of a rotating drum. The drum rotated with 40 revolutions per minute. The time the animal spent on rotating drum was recorded. One pretraining trial was given to each animal. Three more consecutive trials were given to complete the experiment. The average time of these trials was obtained by using Sunyer et al.'s [12] methods.

### 2.5. Open Field (OF)

A video camera (XPod-058, China) attached with computational tracking system, Any-Maze (Stoelting, USA), was used for observing the mice in a chamber which was 40 cm × 40 cm long and walls were 70 cm high. Ten-minute time was used for observation. For each trial the individual mouse was released in the center of the box. Distance covered, mean speed, maximum speed, time mobile, resting time, rotations, and freezing time were studied following Weitzdoerfer et al. [13].

### 2.6. Morris Water Maze (MWM)

Mice were trained to swim towards platform which was hidden under the water 1.5 cm deep. By using compass North East, North West, South West, and South East locations were allocated to the pool which divided the pool into four quadrants. It was attached to computerised tracking/image analyzer system (XPod-058, China) with computational tracking system Any-Maze (Stoelting, USA). During the experiment the centre of the North East quadrant had the platform. 16 training trials were carried out on the mice for four days which is called spatial acquisition phase. Four training trials were taken daily and after each trial there was an interval of 15 minutes. They were allowed to search for platform for 2 minutes.The mice were kept on the platform by hand for 30 sec if they failed to find the platform after 2 min. At the end of acquisition phase, animal was tested for probe trial on the 5th day. Mice started swimming from the south start point. They were allowed maximum time of 60 sec to swim freely. After an interval of six days of the first trial of the retention phase mice received second probe trial for 60 sec. Mice were not given any trial between the 1st and 2nd probe trials according to Sunyer et al. [12]. The swimming pattern during acquisition phase was also recorded.

### 2.7. 2,3,5-Triphenyl-2H-tetrazolium Chloride (TTC) Staining of Brain Slices

Brain of experimental animals was collected from all groups, sliced, and stained with 2,3,5-Triphenyl-2H-*tetrazolium chloride* (TTC) to measure the brain infarcts. Image J software (Australia) was used to measure the infarct volumes (%) in GABA_B_ treated and untreated albino mice.

### 2.8. Statistical Analysis

All the data is expressed as Mean ± Standard deviation. Statistical package Minitab (version 16, Pennsylvania) was used for the analysis of results. Two-sample *t*-test was applied to compare all the studied parameters of open field, rotarod, MWM, and brain infarct volumes between GABA_B_ treated and untreated groups following hypoxia ischemia encephalopathy.

## 3. Results

### 3.1. Rota Rod

**Figure 1:**
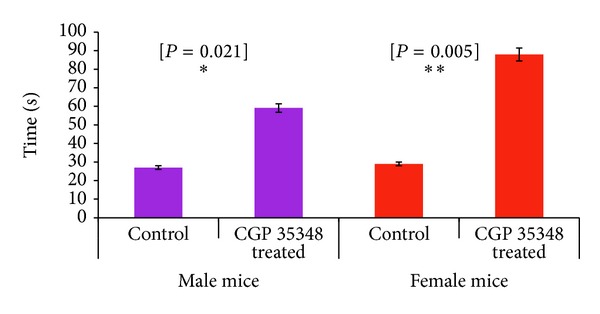
Comparison of rotarod test performance between control and CGP 35348 treated albino mice following hypoxia ischemia encephalopathy. Data is given as mean ± standard error of mean. *P* value indicates the results of two-sample *t*-test. *P* < 0.05 is the least significant (*); *P* < 0.01 is significant (**).

Results of two-sample *t*-test revealed that rotarod test performance was significantly better in CGP 35348 treated albino mice of both genders indicating better neuromuscular coordination than saline treated albino mice following neonatal brain damage (Figure 1). GABA_B_ receptor antagonist treatment male (*P* = 0.005) spent more time on rotating rod than female (*P* = 0.021) albino mice.

### 3.2. Open Field

All studied parameters of open field test remained unaffected when compared between CGP 35348 treated and untreated male albino mice indicating this GABA_B_ receptor antagonist did not affect locomotory and exploratory behavior in these animals following brain damage. Similar analysis in female mice indicated that saline control female remained mobile for longer time (*P* = 0.03) in open field arena as compared to CGP 35348 treated female albino mice indicating a negative effect of treatment on locomotory behavior (Table 1).

### 3.3. Morris Water Maze

#### 3.3.1. Acquisition Phase Results

**Figure 2:**
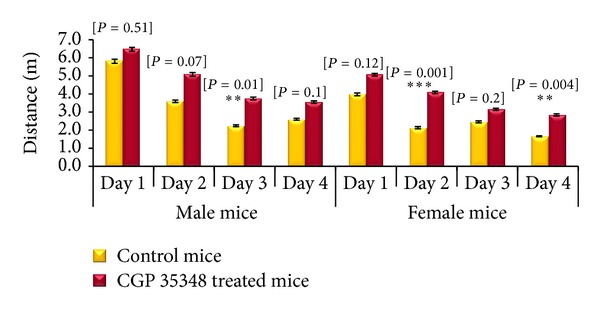
Comparison of distance (m) traveled during acquisition phase between control and CGP 35348 treated male and female albino mice following hypoxia ischemia encephalopathy. Data is given as mean ± standard error of mean. Two-sample *t*-test results expressed as *P* value. *P* > 0.05 is nonsignificant; *P* < 0.01 is significant (**); and *P* < 0.001 is highly significant (***).

Results of two-sample *t*-test for Morris water maze (MWM) acquisition phase revealed that male albino mice treated with CGP 35348 travelled more distance than saline treated control mice throughout the training session but the difference only reached the statistical significance (*P* = 0.01) on training day 3. A similar trend was observed in female albino mice treated with CGP 35348. The difference in distance travelled reached statistical significance on days 2 (*P* = 0.001) and 4 (*P* = 0.004) during acquisition phase following HIE indicating better neuromuscular coordination (Figure 2).

**Figure 3:**
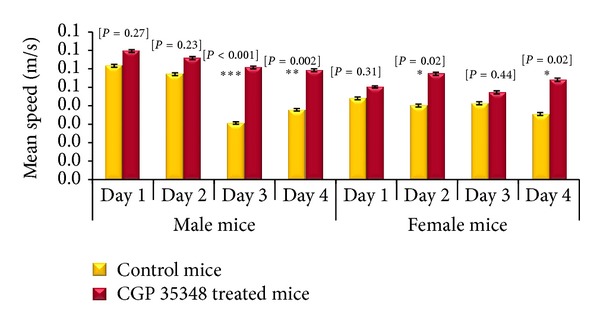
Comparison of mean speed (m/s) during acquisition phase between control and CGP 35348 treated male and female albino mice following hypoxia ischemia encephalopathy. Data is given as mean ± standard error of mean. Two-sample *t*-test results expressed as *P* value. *P* > 0.05 is nonsignificant; *P* < 0.05 is the least significant (*); *P* < 0.01 is significant (**); and *P* < 0.001 is highly significant (***).

A significant effect CGP 35348 treatment on mean swimming speed of male albino mice was observed during training days 3 (*P* = 0.001) and 4 (*P* = 0.002) of acquisition phase as these animals swam with higher speed than their saline treated control group (Figure 3). A similar trend was observed in female albino mice as GABA_B_ receptor antagonist treated female swam with significantly high speed on days 2 (*P* = 0.02) and 4 (*P* = 0.02) of acquisition phase as compared to saline treated control female albino mice (Figure 3).

### 3.4. Retention Phase Results

**Figure 4:**
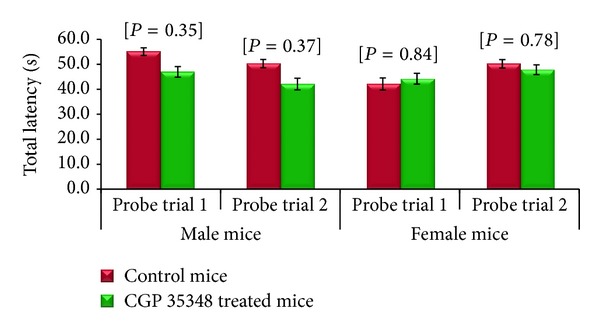
Comparison of total latency (sec) during retention phase between control and CGP 35348 treated male and female albino mice following hypoxia ischemia encephalopathy. Data is given as mean ± standard error of mean. Two-sample *t*-test results expressed as *P* value. *P* > 0.05 is nonsignificant.

Retention phase result revealed that total latency to reach platform area by male albino mice treated with CGP 35348 was less than their control mice group but the value did not reach the statistical significance during probe trials 1 and 2. On the other hand, female albino mice were not affected by CGP 35348 treatments in both probe trials following HIE (Figure 4).

Analysis of parameter mean speed during retention phase revealed that mean speed increased in both probe trials in male and female treated with CGP 35348 as compared to their control groups but the difference did not reach the statistical significance (data not shown here).

### 3.5. Swimming Strategies

**Figure 5:**
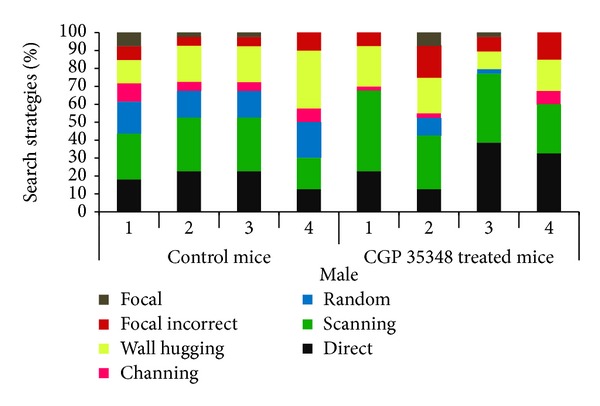
Comparison of swimming strategies during acquisition phase of MWM between control and CGP 35348 treated male albino mice following hypoxia ischemia encephalopathy.

**Figure 6:**
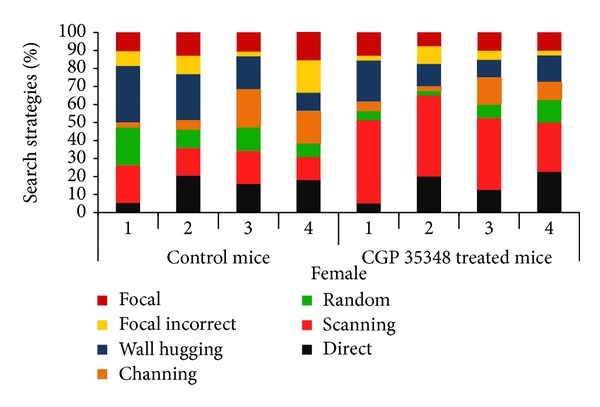
Comparison of swimming strategies during acquisition phase of MWM between control and CGP 35348 treated female albino mice following hypoxia ischemia encephalopathy.

Comparison of swimming strategies indicated that male albino mice treated with CGP 35348 showed better learning, following hypoxic ischemic insult, during acquisition phase as their direct approaches to platform and scanning behaviour were improving over time while saline treated males have decreased direct and scanning strategies while random and wall hugging behaviour increased over time indicating poor learning (Figure 5). Female albino mice showed markedly improved learning as compared to males. CGP 35348 treated female had increased direct, scanning, and focal search approaches for platform indicating better learning while saline treated control females despite having increased direct and focal approaches to platform, they had also increased channing and focal incorrect approaches indicating poor learning (Figure 6).

### 3.6. Histological Studies of Albino Mouse

Brain infarct volumes (% of total brain volume) were studied in comparison between brain slices of male and female albino mice treated with CGP 35348 with their saline treated control groups following HIE. It was observed that the infract size produced in saline treated male and female was not significantly different than the % infract size observed in CGP 35348 treated male (*P* = 0.3) and female (*P* = 0.2) albino mice, respectively. It was observed that female albino mice developed larger infarcts than male mice following hypoxic ischemic insult (Table 2).

## 4. Discussion

GABA_B_ receptor inhibition is known to be associated with the process of learning and memory formation but there is no information available, to date, regarding the application of GABA_B_ receptor antagonists on learning and memory enhancement following HIE. The present study was designed to determine the gender specific effects of CGP 35348 on neuromuscular coordination, exploratory behavior, learning, and memory formation in albino mice following hypoxia ischemia mediated brain damage at postnatal day 10. GABA_B_ receptors present on the pre- and postsynaptic membranes are different from one another in their structure and functions [14]. High concentrations of CGP 35348 can block postsynaptic GABA_B_ receptors but in the neocortex presynaptic GABA_B_ receptors are only affected by CGP 55845 [15]. CGP 35348 acts presynaptically in hippocampal slices [16], which shows that pre- and postsynaptic GABA_B_ receptors differ in the neocortex from each other, and also presynaptic GABA_B_ receptors interneurons differ between various structures in the central nervous system [17].

Our results indicated that GABA_B_ receptor antagonist treated mice performed better on rotating rod as compared to their saline treated control groups. There was a gender specific effect in our experiments and it was observed that CGP 35348 was more effective in female albino mice following hypoxic ischemic insult. Gillani et al. [18] have reported similar gender specific observations in albino mice. They have reported that female albino mice intraperitoneally injected with CGP 35348, GABA_B_ receptor antagonist, spent more time on rotating rod than male albino mice. Various animal studies have demonstrated that agonists of GABA_B_ receptor can decrease body temperature and muscular tension and suppress locomotor activity and also can cause memory deficits [19, 20]. By using the CGP 35348 we have observed the expected results, that is, improved neuromuscular coordination in GABA_B_ receptor antagonist treated albino mice. The gender specific differences are probably due to different effect of hypoxic ischemic insult in both genders.

The open field test measures activity while exploring a novel environment and also tests for cerebral structural integrity [21–23]. Partyka et al. [24] had also reported that GABA_B_ receptor antagonist had very little or no effect on the fundamental mouse behaviors. Our open field test results again had gender specific results. There was no significant effect on exploratory and locomotory behavior of male albino mice treated with GABA_B_ receptor antagonist following HIE but on the other hand female albino mice treated with CGP 35348 exhibited poor exploratory and locomotory behavior as compared to their respective saline treated control groups. Similar results were observed by Gillani et al. [18] when they applied CGP 35348 in male and female albino mice and reported that the application of CGP 35348 resulted in decreased mobility and rotations in female as compared to male albino mice.

Morris water maze is a commonly used behavioral test to compare spatial learning and memory between various treatments and also assess spatial learning and memory as a function of the hippocampus [12, 18, 25, 26]. Our results during the acquisition and retention phase of MWM revealed that CGP 35348 increased the spatial learning in male albino mice as compared to female albino mice following HIE. CGP 35348 also increased the mean speed in male and female albino mice. Swimming strategies used by albino mice also explained that CGP 35348 had better effect on learning in male albino mice following HIE. A similar effect of CGP 35348 on spatial learning was observed by Gillani et al. [18] in albino mice. They reported that CGP 35348 treatment led to the improved learning during the acquisition phase and it also significantly improved memory formation during probe trial in male albino mouse. Nakagawa et al. [27] had reported that GABA_B_ receptor affects cognitive performance in rodents. Behavioral work ranging from memory facilitation to impairment achieved by GABA_B_ receptor blockade [28–32]. Concentration of GABA_B_ receptor antagonist is very crucial to suppress or facilitate the LTP. Inhibition of postsynaptic GABA_B_ receptor can enhance the period of dendritic NMDA receptor-mediated currents which help in the induction of LTP, while inhibition of presynaptic receptors results in self-inhibition of GABA release which did not facilitate the induction of LTP [33].

2,3,5-Triphenyltetrazolium chloride (TTC) is commonly used for the assessment of lesion size in rodent brains after brain damage [34]. By using TTC infarct areas measured always closely resemble those measured with other histological methods [35]. Quantitative assessments of infarct volume by using TTC have proven useful in measuring the volume of brain injury in stroke experimental models and in determining the potential neuroprotective agents [36].

Histological results indicated that there was no effect of GABA_B_ receptor antagonist on infracts size indicating that both of these chemicals were not playing any role in decreasing the brain lesion size in both genders. Our result indicated that female albino mice were more susceptible to HIE than males as their infarct size was larger than male albino mice. This could possibly be the reason why female albino mice did not perform well in rotarod, open field, and Morris water maze test following hypoxic ischemic encephalopathy.

Various studies have demonstrated major physiological differences in male and female neurons when grown separately in cell culture [37, 38] but the mechanisms for these sex differences are poorly understood. For example, cerebral cortical electrical activity matures earlier in male than in female term neonates [39]. Recently Zhu et al. [40] have confirmed that sex specific differences in cell death pathways are also found in an in vivo model of hypoxia ischemia in mice and probably account for gender specific brain damage observed in present studies.

In summary, we used CGP 35348 to observe its effect on behavior and physiology of male and female albino mice following brain damage. We observed that overall CGP 35348 had improved the motor function in male and female albino mice but this treatment was more effective in females than in male albino mice. In open field test, female albino mice displayed poor exploratory and locomotory behavior following CGP 35348 supplementation. During Morris water maze test, we again observed the gender specific effects as CGP 35348 improves spatial learning and memory in male albino mice but had no effect on female albino mice following HIE indicating that CGP 35348 has a potential to improve neuromuscular coordination and spatial learning in male albino mice.

## Figures and Tables

**Table 1:** Comparison of open field test parameters between CGP 35348 and saline treated male and female albino mice following hypoxia ischemia encephalopathy.

Parameters	Control male mice(*N* = 10)	CGP 35348 treated male mice(*N* = 10)	*P* value	Control female mice (*N* = 10)	CGP 35348 treatedfemale mice(*N* = 10)	*P* value
Distance (m)	19.3 ± 0.6	18.7 ± 0.6	0.83	29 ± 1	18 ± 1	0.07
Mean speed (m/s)	0.03 ± 0.00	0.03 ± 0.00	0.82	0.05 ± 0.00	0.03 ± 0.00	0.08
Time mobile (sec)	436 ± 7.5	461 ± 5.9	0.42	518 ± 5	412 ± 10	0.03*
Time immobile (sec)	163 ± 7.5	139 ± 5.9	0.42	82 ± 5	188 ± 10	0.03*
Mobile episodes	32.4 ± 0.6	32.9 ± 0.9	0.89	21 ± 1	34 ± 1	0.07
Immobile episodes	31.6 ± 0.6	32.0 ± 0.9	0.91	20 ± 1	34 ± 1	0.06
Max. speed (m/s)	0.24 ± 0.01	0.25 ± 0.00	0.71	0.29 ± 0.01	0.25 ± 0.01	0.59
Rotations	21.4 ± 0.5	24.5 ± 0.5	0.22	36 ± 1	24 ± 1	0.18
Clockwise rotations	12.4 ± 0.3	12.4 ± 0.3	1.0	17 ± 1	11 ± 1	0.41
Anticlockwise rotation	9.0 ± 0.3	12.1 ± 0.4	0.90	18 ± 1	12 ± 1	0.09

Data is given as mean ± standard error of mean. *P* value indicates the results of two-sample *t*-test. *P* > 0.05 is nonsignificant; *P* < 0.05 is the least significant (∗).

**Table 2:** Comparison of % infarct volume between CGP 35348 treated male and female albino mice and their saline treated control group following hypoxia ischemia insult.

Parameter	Male albino mice		*P* value	Female albino mice		*P* value
	Control (*N* = 10)	CGP 35348 treated (*N* = 10)		Control (*N* = 10)	CGP 35348 treated (*N* = 10)	
% Infarct volume	20.0 ± 0.1	24 ± 0.1	0.30	30 ± 0.1	26 ± 0.1	0.20

Data is given as mean ± standard error of mean. Two-sample *t*-test results expressed as *P* value. *P* > 0.05 is nonsignificant.
